# The Utrecht Work Engagement Scale for Students (UWES–9S): Factorial Validity, Reliability, and Measurement Invariance in a Chilean Sample of Undergraduate University Students

**DOI:** 10.3389/fpsyg.2019.01017

**Published:** 2019-04-30

**Authors:** Marcos A. Carmona-Halty, Wilmar B. Schaufeli, Marisa Salanova

**Affiliations:** ^1^Escuela de Psicología y Filosofía, Universidad de Tarapacá, Arica, Chile; ^2^Research Unit of Occupational & Organisational Psychology and Professional Learning, KU Leuven, Leuven, Belgium; ^3^Department of Social, Health and Organizational Psychology, Utrecht University, Utrecht, Netherlands; ^4^WANT Research Team, Universitat Jaume I, Castellón de la Plana, Spain

**Keywords:** UWES, psychometric analyses, academic engagement, university students, invariance across gender

## Abstract

This brief report examines the within–network construct validity of the UWES–9S in a convenience sample of 1502 Chilean students (52% were female) ranging between 18 and 25 years old. The results of confirmatory factor analysis supported a solution with three related factors that fit significantly better than a one-factor solution. The three subscales (i.e., vigor, dedication, and absorption) and the overall UWES–9S showed satisfactory internal consistency. The results of multiple–group confirmatory factor analysis supported gender invariance. Overall, the UWES–9S was found to be a reliable and valid scale to assess academic engagement in Chilean undergraduate university students.

## Introduction

The concept of work engagement –a positive, fulfilling, work related state of mind characterized by vigor, dedication, and absorption (Schaufeli et al., 2002b) – refers to the work activities employees perform. However, this construct has also been applied to the activities students perform, referred to as academic engagement (Schaufeli et al., 2002a). The reasoning is that, psychologically speaking, the activities students perform can also be considered as “work,” in the sense of goal–directed and structured activities, which are compulsory in nature. More specifically, *vigor* refers to high levels of energy and mental resilience while studying, the willingness to invest effort in studying, and persistence even when encountering difficulties; *dedication* refers to being strongly involved in one’s studies and experiencing a sense of significance, enthusiasm, inspiration, pride, and challenge; and *absorption* refers to being fully concentrated and happily engrossed in what one is studying, where time passes quickly and one finds it difficult to detaching him/herself from studying (Schaufeli, 2017).

After almost two decades of research, we now know that academic engagement is associated with personality traits (Sulea et al., 2015), personal resources (Siu et al., 2014), and positive emotions (Ouweneel et al., 2011). Moreover, it has positive consequences for students, such as greater involvement in their studies (Loscalzo and Giannini, 2018), higher levels of wellbeing (Tayama et al., 2018), and better academic performance (Salanova et al., 2003, 2010).

The research on academic engagement carried out so far is at pre-professional levels (i.e., undergraduate university students) and uses the Utrecht Work Engagement Scale for students (UWES–S; Schaufeli et al., 2002a). The UWES–S is the student version of the most widely used instrument to assess work engagement, the Utrecht Work Engagement Scale (UWES; Schaufeli et al., 2002b). Originally, the UWES included 17 items and three dimensions (i.e., vigor, dedication, and absorption). Later, for pragmatic reasons, the UWES was reduced, resulting in a 9–item version (UWES–9) that showed adequate psychometric properties as well (Schaufeli et al., 2006).

The UWES–9 has been validated in industrial–organizational settings in various countries (e.g., Brazil–Sinval et al., 2018; Finland–Seppälä et al., 2009; Italy–Balducci et al., 2010; Japan–Shimazu et al., 2008; Norway–Nerstad et al., 2010; Russia–Lovakov et al., 2017; Serbia–Petrovic et al., 2017; and South Africa–Storm and Rothmann, 2003; among others–Schaufeli et al., 2006). However, validity studies on the UWES–9S are still scarce with mixed evidence for its three–factor structure (Sánchez–Cardona et al., 2016; Loscalzo and Giannini, 2018). However, it cannot be ruled out that this is caused by flaws in sampling and research design (e.g., Parra and Pérez, 2010; Portalanza–Chavarria et al., 2017).

The UWES–9S is also popular in Spanish–speaking South American countries, where no rigorous psychometric evaluation has been carried out so far. Thus, aim of the present brief report is to fill this gap by examining the factorial validity, reliability, and gender invariance of the UWES–9S in a sample of undergraduate Chilean university students. Providing empirical evidence about the UWES–9S makes it possible to examine the continuum from academic to working life, which has hardly been addressed (Upadyaya and Salmela–Aro, 2013).

Based on the arguments presented, we hypothesize the following: The UWES–9S will demonstrate acceptable psychometric properties in a Chilean sample of undergraduate university students.

## Materials and Methods

### Participants

The sample consisted of 1502 Chilean undergraduate university students, aged between 18 and 25 years (*M* = 20.55; SD = 1.91; 48% male and 52% female). The students came from different fields of study: social sciences (34%; include law, psychology, and Anthropology), health sciences (36%; include Nursing and Medicine), and natural sciences (30%; include Physics, Math, and Chemistry). Of the 1502 participants, 23% were in the first year, 34% in the second year, 26% in the third year, 16% in the fourth year, and 7% in the fifth year of their studies. Finally, 15% correspond to low, 80% to medium, and 5% to high socio–economic levels.

### Procedure

The data were collected in the context of a research project about the role of students’ well–being in academic achievement. The project was approved by the research ethics committee of the host university. The participants voluntarily filled out an online questionnaire during their usual class schedule.

### Instrument

The UWES–9S (Schaufeli et al., 2006) is a nine–item self–report scale grouped into three subscales with three items each (see Table 1): vigor (VI), dedication (DE), and absorption (AB). All items are scored on a seven–point frequency rating scale ranging from 0 (*never*) to 6 (*always*). For the purposes of this study, the Spanish version of the UWES–9S^1^ was adapted slightly –following the guidelines of the International Test Commission for adapting tests across cultures (Muñiz et al., 2013)– in order to improve its comprehension by undergraduate Chilean students. Furthermore, before the data collection, the nine items were piloted in a small group of participants to verify their clarity (*n* = 10). None of the participants expressed problems with understanding the items or the answering format of the UWES–9S.

**TABLE 1:** Descriptive information of the UWES–9S and factor loadings resulting from CFA.

	Mean (SD)	S	K	Factor loadings^1^			Reliability	
							α index if item	ω index if item
				VI	DE	AB	is dropped	is dropped
(1) When I’m doing my work as a student, I feel bursting with energy.	3.41 (1.53)	−0.265	−0.358	0.78**			0.887	0.889
(2) I feel energetic and capable when I’m studying or going to class.	3.08 (1.72)	−0.121	−0.775	0.76**			0.887	0.889
(3) I am enthusiastic about my studies.	3.93 (1.59)	−0.467	−0.424		0.73**		0.889	0.891
(4) My studies inspire me.	4.01 (1.53)	−0.507	−0.298		0.70**		0.892	0.893
(5) When I get up in the morning, I feel like going to class.	3.33 (1.73)	−0.266	−0.717	0.76**			0.889	0.890
(6) I feel happy when I am studying intensely.	3.37 (1.71)	−0.232	−0.711			0.80**	0.886	0.887
(7) I am proud of my studies.	3.39 (1.59)	−0.272	−0.434		0.89**		0.878	0.879
(8) I am immersed in my studies.	3.00 (1.77)	−0.027	−0.854			0.60**	0.898	0.899
(9) I get carried away when I am studying.	3.17 (2.04)	−0.162	−1.177			0.60**	0.897	0.899
				Correlations			α index	ω index
Vigor (items 1, 2, and 5)	3.27 (1.41)	−0.190	−0.540	–			0.808	0.810
Dedication (items 3, 4, and 7)	3.77 (1.35)	−0.482	−0.212	0.73**	–		0.819	0.822
Absorption (items 6, 8, and 9)	3.17 (1.48)	−0.142	−0.679	0.65**	0.66**	–	0.729	0.731
UWES–9S	3.40 (1.25)	−0.239	−0.400	0.89**	0.89**	0.87**	0.900	0.902

SD, standard deviation; S, skewness; K, kurtosis; VI, vigor; DE, dedication; AB, absorption; **p < 0.001; ^1^factor loadings from UWES–9S three–related factor revised solution.

### Statistical Analyses

All analyses were conducted using SPSS AMOS 23.0 (IBM Corp, 2015) and JASP computer software (Jasp Team, 2018). First, we examined the descriptive statistic values, the internal consistency –using Cronbach’s alpha and McDonald’s omega indexes– and gender differences. To examine the factor structure, we performed confirmatory factor analysis (CFA) through the maximum likelihood estimation approach. To evaluate its goodness of fit, we computed the chi–square (χ^2^) and normed χ^2^, root–mean–squared error of approximation (RMSEA) with a confidence interval (90% CI), comparative fit index (CFI), and standardized root mean residual (SRMR). To help evaluate the cut–off and determine model fit, we followed the guidelines published by the European Journal of Psychological Assessment (Schweizer, 2010). To explore gender invariance –following Chen (2008)– we tested for configural (i.e., same structure across groups), metric (i.e., same factor loadings across groups), and scalar invariance (i.e., same intercepts across groups) through multiple–group CFA. To evaluate the magnitude of the change in the fit index, we followed the recommendations of Cheung and Rensvold (2002), who suggested that an absolute difference in CFI (i.e., ΔCFI) or SRMR (i.e., ΔSRMR) of less than 0.01 would indicate that the models are equivalent in terms of fit. In addition, although the chi–squared difference test may overstate non-substantive discrepancies –because it is sensitive to sample size– we also report this value.

## Results

### Preliminary Analysis and Reliably of the Scores

As Table 1 shows, the skewness of the score distribution of the UWES–9S items ranged between −0.027 and −0.507, and kurtosis values ranged between −0.298 and −0.117. According to Finney and DiStefano (2006), these values indicate that the assumption of normality has not been violated. In addition, the size of the correlations ranged between 0.65 and 0.89, revealing strong significant relationships between the subscales of the UWES. Independent–sample *t*-tests revealed that –in accordance with previous studies (Schaufeli et al., 2017)– female students (*M* = 3.510, SD = 1.253) scored significantly higher than male (*M* = 3.315, SD = 1.258) students, *t* (1499) = 3.007, *p* < 0.05, *d* = 0.155, 95% IC (0.054, 0.257). However, based on Cohen’s (1988) criterion, the effect size was small. Finally, the analysis of Cronbach’s alpha and McDonald’s omega indexes if an item is deleted allowed us to retain the nine items and obtain good internal consistencies for the subscales and the overall UWES–9S (Table 1).

### Sources of Validity Evidence of Internal Structure

Based on previous research on the validity of the UWES (e.g., Schaufeli et al., 2006, 2017), we tested two models: one that assumes that all the engagement items load in one single factor (M1), and one that proposes three related factors (M2). The results of the CFAs show that the data fit of both models to the data is good, except that the RMSEA value exceeds the criterion of 0.08 (see M1 and M2 in Table 2). To decide whether the model needed re–specification, we inspected the modification indexes, which indicated that allowing the errors for two items from the absorption subscale to correlate could increase the model fit (items 8 and 9, *r* = 0.30, *p* < 0.005). The two resulting models (see M3 and M4 in Table 2) fitted the data significantly better (see Table 2, M1–M3 for the one–factor solution and M2–M4 for the three–factor solution). In addition, the comparison of the re–specified models shows that the three–factor model (M4) fits significantly better than the re–specified model with one factor (M3). Hence, M4 was used as the baseline model in the multiple–group CFA.

**TABLE 2:** Fit indexes for single–group and multiple–group CFA.

	χ^2^	*df*	χ^2^/*df*	RMSEA	90% CI	CFI	IFI	SRMR	CMs	Δ χ^2^(Δ *_df_*)	Δ CFI	Δ SRMR
**Single–group CFA**												
M1 one factor	410.061**	27	15.187	0.097	[0.089,0.106]	0.943	0.943	0.041				
M2 three–factors	282.862**	24	11.786	0.085	[0.076,0.094]	0.962	0.962	0.034				
M3 one factor revised	261.884**	26	10.072	0.078	[0.069,0.086]	0.965	0.965	0.027	M1–M3	148.177 (1)**	0.022	0.014
M4 three–factors revised	159.679**	23	6.943	0.063	[0.054,0.072]	0.980	0.980	0.021	M2–M4	123.183 (1)**	0.018	0.013
**Multiple–group CFA^1^**									M3–M4	102. 205 (3)**	0.015	0.006
M5 configural invariance	186.522**	46	4.055	0.045	[0.038,0.052]	0.979	0.979	0.022	–	–	–	–
M6 metric invariance	187.606**	52	3.608	0.042	[0.035,0.048]	0.980	0.980	0.022	M5–M6	1.084 (6)ns	0.001	0.000
M7 scalar invariance	202.574**	61	3.321	0.039	[0.033, 0.045]	0.979	0.979	0.022	M6–M7	16.052 (15)ns	00.001	0.000

**p < 0.001; ^1^invariance between male and female students; $\chi^{2}$, Chi-square; *df*, degree of freedom; RMSEA, root mean square error of approximation; CI, 90% confidence interval; CFI, comparative fit index; IFI, incremental fit index; SRMR, standardized root mean square residual; CMs, comparisons between models.

### Measurement Invariance Across Gender

The baseline model showed an acceptable fit, with support for configural invariance (see M5 in Table 2). In the next step, equality constraints were imposed on all factor loadings in order to examine metric invariance. The resulting model (M6 in Table 2) also achieved an acceptable fit. When comparing M5 and M6, the change in the chi–square difference test was not statistically significant. In addition, the absolute difference in CFI and SRMR was less than 0.001. Thus, we concluded that metric invariance across genders is supported. Next, equality constraints were imposed on all intercepts to test scalar invariance. This model (M7 in Table 2) also achieved an acceptable fit. Therefore, following the same reasoning described above, we concluded that scalar invariance across genders is supported. Taken together, we concluded that academic engagement has the same meaning in our male and female participants.

## Discussion

Limited attention has been paid to examining the psychometric properties of the UWES–9S, particular in Spanish–speaking Latin American countries. Therefore, this brief report provides empirical evidence about the factor validity, reliability, and measurement invariance across male and female Chilean students.

Our results coincide with international research on the factor structure of the UWES–9 (Schaufeli et al., 2002a, b, 2006; Balducci et al., 2010; Sánchez–Cardona et al., 2016; Loscalzo and Giannini, 2018; Tayama et al., 2018). In other words, vigor, dedication, and absorption are satisfactorily explained by a solution with three related factors. In addition, reliability analysis –based on Cronbach’s alpha and McDonald’s omega indexes– of the subscales and the overall UWES–9S indicated high internal consistency. Gender invariance was also demonstrated, revealing its capacity to evaluate academic engagement in a similar way and with the same precision in male and female undergraduate university students. Together, these results lead us to conclude that the UWES–9S produces scores that could be of value, in terms of reliability and validity, in measuring academic engagement in Chilean undergraduate university students.

The main strength of the present study is the large sample used. However, there are also some limitations that should be mentioned. First, we used a convenience sample that does not represent all Chilean undergraduate university students adequately, or all academic fields. Thus, a more representative and diversified sample is needed to compare our results. Second, we only focused on a within–network validity approach. Hence, a nomological network approach is needed to explore significant relationships between the UWES–9S and other study–related variables, such as academic burnout, psychological capital, or academic performance, to test convergent and predictive validity. Third, modification indices have been used to allow two errors to correlate in order to improve the fit of the UWES–9S. This strategy is coherent with previous UWES research (e.g., Schaufeli, 2017; Loscalzo and Giannini, 2018), and it is justified by overlapping meaning or item content (MacCallum et al., 1992): “I am immersed in my studies” and “I get carried away when I am studying.”

In order to expand the future research on academic engagement, it would be interesting to examine the applicability of the UWES–9S at levels that have not yet been explored (e.g., class–level academic engagement). In addition, examining the predictive role of the UWES–9S –as an external report (e.g., peer rated by teachers and parents)– in students’ academic outcomes could be considered a fruitful future line of research. Finally, we want to mention two challenges that would help to increase the knowledge about the UWES in academic contexts. The first would be to examine the equivalence (i.e., measurement invariance) of the UWES–9S across countries, and the second to establish cut–off scores for high and low levels of academic engagement.

## Ethics Statement

This study was carried out in accordance with the recommendations of Ethics Committee of the Universidad de Tarapacá, with written informed consent from all subjects. All subjects gave written informed consent in accordance with the Declaration of Helsinki. The protocol was approved by Research Ethics Committee of the Universidad de Tarapacá.

## Author Contributions

All authors contributed equally to the research design and wrote the manuscript.

## Conflict of Interest Statement

The authors declare that the research was conducted in the absence of any commercial or financial relationships that could be construed as a potential conflict of interest.

## Footnotes

1 www.wilmarschaufeli.nl
